# A Typology of Scientific Advisory Committees

**DOI:** 10.1002/gch2.201800004

**Published:** 2018-06-19

**Authors:** Gaëlle M. N. Groux, Steven J. Hoffman, Trygve Ottersen

**Affiliations:** ^1^ Global Strategy Lab York University/University of Ottawa Ontario Canada; ^2^ Faculty of Common Law University of Ottawa Ottawa, ON K1N 6N5 Canada; ^3^ Dahdaleh Institute for Global Health Faculty of Health and Osgoode Hall Law School York University Toronto M3J 1P3 Canada; ^4^ Department of Global Health and Population Harvard TH Chan School of Public Health Harvard University Boston MA 02138 USA; ^5^ Division for Health Services Norwegian Institute of Public Health Oslo 0403 Norway; ^6^ Oslo Group on Global Health Policy Department of Community Medicine and Global Health and Centre for Global Health University of Oslo Oslo 0318 Norway

**Keywords:** evidence‐based decision making, scientific advisory committee designs, scientific advisory committees, typology of scientific advisory committees

## Abstract

The era of evidence‐informed decision‐making has seen increased use of the scientific advisory committee (SAC) to provide decision‐makers with scientific advice, despite limited evidence of the effectiveness or best strategies for designing these committees. In this study, an in‐depth review of academic and gray literature is undertaken to outline the global landscape of SACs. The development of a typology is also undertaken that categorizes SACs along six dimensions: 1) sector, 2) level of operation, 3) permanence, 4) target audience, 5) autonomy, and 6) nature of advice. It is found that SACs differ profoundly in each of these dimensions and provide examples demonstrating this variation. The landscape and typology can help decision‐makers understand the key elements of SAC design and reform, and the results will also inform future research on the design and effectiveness of SACs. With SACs expected to promote evidence‐informed decision‐making, it is imperative that the design of these committees themselves is guided by evidence.

## 1 Introduction

In the era of evidence‐informed decision‐making policy makers increasingly seek scientific advice via scientific advisory committees (SACs). These committees are one of several institutional arrangements that governments and organizations use, based on the simple but powerful premise that pressing policy choices should be informed by the best available science. There is a fast‐growing scholarly literature^1^, ^2^, ^3^ that underscores the complex role that scientific research plays in the policy process. And yet, the actual relationship between science and policy is far from settled, with SACs introducing an additional layer of complexity in the science‐policy nexus.

Increasingly, these committees have become widely used;^4^, ^5^ for example, the World Health Organization (WHO) held 47 expert advisory panels in 2014, in addition to numerous expert committees, ad hoc advisory groups, and scientific groups. Similarly, the United States government engaged in 916 federal advisory committees in 2006, while^6^ these and other SACs are intended to support decision‐making by acting as mediators between researchers and policymakers,^7^ and by gathering and summarizing vast amounts of evidence pertaining to the policy question at hand.^8^ Through researcher participation, SACs may also confer legitimacy on the decision‐making process and the final decision.^9^

Despite the importance of SACs–in terms of their sheer number as well as the roles they are expected to play–the study of these committees is still at an early stage. There is an extensive literature on scientific advice, but only a limited literature specific to SACs.^10^ Thus, while SACs come in many shapes and sizes, we have little knowledge about the design of an effective SAC. Recent criticism of several high‐profile SACs has highlighted this concern; for example, committees affiliated with the Intergovernmental Panel on Climate Change (IPCC) and the WHO have been criticized in the past for lack of independence.^11^

In this study, a SAC, appointed by either governments and/or civil society organizations, is defined as a) a group of individuals with relevant expertise; b) where the group provides advice to decision‐makers; and c) the advice is based predominantly on research evidence from the natural or social sciences. The terminology used to describe SACs or similar bodies differs widely. Among the many terms that may refer to SAC as defined here include “expert committee,” “scientific panels,” and “technical advisory group”. We maintain that the effectiveness of a SAC increases with the quality of its advice, the relevance of the advice to decision‐makers, and the legitimacy of the advice and the preceding process.^12^

Few systematic characterizations of the complex and evolving landscape of SACs exist in the current body of literature. Furthermore, typologies that can facilitate the study of the institutional design of SACs are in short supply. **Table** **1** describes and compares the three comprehensive typologies known to us. These typologies differ in their purpose, focus, and categorization of SACs.^64^, ^65^, ^66^ To our knowledge, no comprehensive typology exists that addresses SACs across all sectors and levels and whose primary purpose is to facilitate better understanding of what institutional designs make SACs most effective; our goal in this article is to describe such a typology.

**Table 1:** Previously proposed typologies of scientific advisory committees

Aspects of typology	Level of operation	Geographical scope	Broad categories studied	Focus
Glynn et al.^64^	National, Regional	European: EU15 states and EU itself	General: users, policy areas, and status of advisory bodies Structural: secretariat and membership of advisory bodies Functional: Scope of work, independence, transparency, generation, delivery and responses of advice, changes in the advisory system	To systematically map and characterize significant scientific advisory bodies in Europe
Schulz et al.^65^	National	European: France, UK, Germany, Sweden, Netherland	Configuration: size and temporal orientation Administration: regulation, financing and obligations for government Composition: how advisory systems are manned Political‐administrative regimes	Identifying institutional elements of advisory systems and their political‐administrative regime setting
Heinrichs^66^	National	Germany and the US	Distance from Politics Policy Function Dealing with Pluralism of Knowledge, Values and Interests Communication, Interaction, Inclusion	Orientation tool for assessment and optimization of advisory structures

Decision‐makers must make choices when establishing or reforming SACs; a broad overview and typology of SACs can help decision‐makers better understand these options. A landscape of SACs helps researchers and policymakers understand the wide variety of designs that SACs have taken in different contexts, and a typology can help researchers when studying the design of SACs and particularly when examining what institutional features make them effective. It is important to have a strong understanding of the institutional design of SACs as these design features are those over which policy‐makers have control. There are also certain contextual factors that interact with design features, and which need to be understood prior to designing an SAC. It is important therefore to provide policy and decision‐makers with the tools and evidence to design effective SACs in their particular context.

While there is a literature on the environmental factors affect SACs, it is key to have a strong understanding of the institutional design of SACs. These are the features over which decision‐makers have control, and it is therefore important to provide them with tools and evidence to design effective SACs. The objective of this study was to describe the global landscape of SACs and to develop a typology that can both inform the design of SACs and facilitate future studies on effective SAC design.

## 2 Typology

We propose a typology that based on six characteristics of SACs (see **Table** **2**) namely: 1) the sector in which the SAC operates; 2) the level of jurisdiction at which the SAC operates; 3) the degree to which the SAC is permanent; 4) the degree of autonomy with which the SAC works; 5) the target audience of the advice; and 6) the nature of advice. Our proposed typology differs from those outlined in Table 1, but integrates key elements from each. Each characteristic and its role in the typology are further described below.

**Table 2:** Proposed typology of scientific advisory committees

Characteristics	Options for the characteristics
Sector	Health Environment Education National security Justice Energy and transportation Other
Level of operation	Supranational National Subnational
Permanence	Time‐limited Standing
Target audience	Internal External
Autonomy	Arms‐length Embedded
Nature of advice	Descriptive Prescriptive

## 3 Global Landscape

The proposed typology serves two functions: first, the typology can help structure the description of the landscape itself, and second, applying the typology to existing SACs can help illustrate the potential role of each characteristic in the design of SACs.

### 3.1 Sector

SACs are used in a wide variety of fields and are commonly seen in the areas of health, food, and nutrition, and the environment.^13^ However, SACs also exist in the fields of education,^14^ law,^15^ trade,^16^ human rights,^17^ and national security.^18^

In health policy, the uses of SACs have included evaluating the safety of medications^19^ and providing general health advice to the general public.^20^ For example, Health Canada, the main federal department for health regulations and advice, has at least 20 external advisory bodies to advise it on issues, such as cancer treatments, opioids, medical devices used in the cardiovascular systems, and traditional Chinese medicine.^21^

Environmental SACs have also been used to advise governments on diverse topics including land conservation and climate change. For example, the German government has established many permanent SACs that focus on national and European environmental policies, international environmental and development policy, land regulations and policies.^22^

SACs also play a role in national defense policy. For example, the US Department of Defense (DoD) has relied on the Defense Science Board since 1956 for advice on issues, such as nuclear weapon surety, cyber deterrence, military satellite communication, and unmanned undersea devices.^23^ The board is composed of scientists in the fields of technology, science, manufacturing, and acquisition processes, and provides advice and recommendations to the DoD's scientific and technical enterprise.^24^

The relationship between the design and effectiveness of SACs may differ across sectors, making sector an important characteristic for consideration. For example, attitudes toward evidence and quality of evidence differ between fields, and these cultural differences may affect how a SAC is constructed. Similarly, SACs are more common in environmental policy making than in other sectors than others.

### 3.2 Level of Operation

SACs exist at all levels of jurisdiction: international, national, and subnational. They have been used by both governmental and nongovernmental entities, including charities, corporations, and nongovernmental organizations.^25^ Most literature on SACs focuses on those affiliated with national governments; we describe examples of these SACs later in this article.

At the international level, SACs are often set up to provide advice to national governments. One example is the European Academies Science Advisory Council (EASAC), which provides advice in the areas of energy, environment, and biosciences to national governments in the European Union (EU).^26^ However, SACs at the international level may also provide advice to international organizations such as the United Nations Environmental Program, to name but one.^27^

SACs at the subnational level may be affiliated with state or municipal jurisdictions. One example is the Puget Sound Water Quality Authority, which was a 21‐expert advisory committee that advised the state Governor on the environmental degradation of Puget Sound^28^ after concerns were raised over sewage, dead whales, and dumping of dredge spoils.^29^

### 3.3 Permanence

SACs differ dramatically in their longevity. While some SACs are ad hoc, established to address a single question for a limited period of time, others are standing SACs, which address a series of questions over an extended period of time. The duration of a SAC can vary from days to decades.

A prominent example of long‐standing SAC is the WHO Expert Committee on Selection and Use of Essential Medicines, which has been active for decades. It was established in 1977 to assist WHO member state in identifying essential medicines for their populations.^30^ However, the WHO often uses ad hoc advisory committees as well. This is especially true when an issue requires scientific input but does not warrant an entirely new permanent SAC. For example, in response to the Zika Virus pandemic in 2015 and 2016, WHO convened an advisory group on aircraft disinfection for controlling the international spread of vector‐borne diseases.^31^ This panel ended after its advice was published. Another example of short‐term SACs is emergency committees to determine whether the spread of a disease constitutes a public health emergency of international concern (PHEIR); these committees are generally disbanded after the PHEIR is considered over.^32^

Permanence can impact the effectiveness of SACs in multiple ways. Although temporary and permanent SACs are both able to provide advice that aims to address challenges in the long term,^33^ some argue that ad hoc committees tend to be more useful for advice on “hot” crisis or short‐term issues, and permanent bodies tend to orient themselves toward long‐term policy advice.^34^

### 3.4 Target Audience

All SACs aim to provide advice to decision‐makers; a key difference among SACs is whether the target user of the advice is internal or external to the institution that commissioned the SAC. For example, many SACs established by national governments have internal target audiences. For instance, Germany has permanent advisory bodies for almost all federal ministries,^35^ including the German Council for Land Conservation, German Advisory Council on Global Change, the German Advisory Council for the Environment, and many more.^22^ These SACs provide advice to the government on various environmental issues. Similarly, the WHO regularly consults with SACs; one of these is the Guidelines Review Committee, which reviews the WHO's own guidelines.^36^

By contrast, the EASAC which was established by the national science academies of the EU Member States, provides scientific advice to many European governments on issue, such as the environment, energy, and the biosciences.^26^ Some SACs also go beyond providing advice to governments, providing advice directly to knowledge users. For example, the WHO creates a number of SACs that develop and issue, in the name of the WHO, about 200 guidelines per year.^37^

### 3.5 Degree of Independence

The need for SAC independence is a point of contention for many.,^38^, ^39^ Under a broad understanding, a SAC is considered independent to the extent that it is not influenced by outside interests^40^ or nonscientific considerations.^41^ The meaning of independence is quite broad and could include the political or commercial interests of those who fund the SACs, those who have commissioned it, the users of advice, or other powerful actors. For the purposes of this article, we will focus on independence from the users of the advice, such as the organization commissioning the advice, or the organizations that will be using it in the end. When assessing independence, factors that may be considered include the means by which appointment of experts to the panel is made;^42^ the role of commissioners, and the role of users (often policy‐makers) in the selection of issue;^43^ the role of these actors in the generation of advice;^44^ and the flow of communication between the SAC and users in the process of generating advice.^45^ Transparency makes it easier to judge a SAC's level of independence and may also encourage the SAC to become more independent, but transparency is neither a necessary nor sufficient condition for independence.^46^ While independence is generally considered important, it needs to be aligned with other objectives, such as proper regulation and oversight of SACs.^47^ SACs may also have to interact with users and other key factors to ensure that their advice is relevant to decision‐makers.^48^

Many governments and institutions stress that their SACs are independent. One relatively independent, body of SACs is the Canadian Council of Academies (CCA). The Council is funded by the Canadian government and is mandated to do at least five assessments for the federal government every year.^49^ However, the government appoints only 4 of the 12 board members of the CCA, and the government has no interaction with the scientific committees once an assessment is requested. In addition, any sponsor of an assessment is not involved in the selection of committee members and their deliberation, and the assessment undergoes a peer review process.^50^ This balance allows the CCA to ensure relevant advice is given, without having undue influence from the users of the advice during the process of generating an assessment.

On the other end of the spectrum, the IPCC has been criticized for lack of independence. Its members are appointed directly by governments of each country, the users and funders of the advice,^51^ and political representatives are able to contribute at various levels of the deliberations of the IPCC.^52^ In addition, political representatives of the countries have the right to modify or remove parts of IPCC report summaries during and at the end of the process.^53^ Nevertheless, many view the IPCC as a successful scientific advisory committee that has been influential in drawing a consensus around climate change internationally.^54^

Many design choices will affect the independence of the SAC. For example, independence may be compromised if committee members represent government or industry actors and special interests. This, in turn, may reduce the SAC's quality or legitimacy, thereby limiting its effectiveness. At the same time, the level of independence can influence how other design features impact effectiveness. For example, when designing a SAC, government or industry representation in the committee may be required due to political considerations. This may then determine what decision‐making rule will make the SAC most effective. For example, if consensus rather than majority vote is required, government or industry representatives will be able to veto any advice that goes against their interests.

### 3.6 Nature of Advice

SACs can be called upon to generate distinct types of advice. Some SACs provide advice that is almost purely descriptive–describing situations without explicitly attempting to influence behaviour or policy–while other SACs are prescriptive, offering recommendations about what the decision‐maker should do. This type of prescriptive advice, “exploits some of the descriptive theories and empirical findings of descriptive studies” to add actionable advice.^55^

One SAC that offers descriptive advice is the Parliamentary Office of Science and Technology.^56^ This UK body provides summaries for parliamentarians, the purpose of which is to provide “balanced and accessible overviews of research from across the biological, physical, and social sciences, and engineering and technology”.^57^ Rather than suggesting what policy is the best, these papers are meant to act as the basis for parliamentary deliberations. An example of a more prescriptive SAC is the European Commission's High Level Group of Scientific Advisors, which is specifically requested to provide policy‐makers with scientific advice on specific policy issues.^58^

Whether a SAC provides descriptive or prescriptive advice is a choice often made when designing and commissioning the SAC. The nature of the SAC's advice can influence its relevance for decision‐makers and its legitimacy, and therefore the effectiveness of the SAC. For example, if the advice does not provide direction on policy, decision‐makers may find the advice of limited relevance. One study looking at advice given to individuals and finds that individuals found information most useful when it was prescriptive.^59^ However, if the advice is overly prescriptive, the SAC may be perceived as trespassing on the domain of the decision‐makers, and the SAC may lose legitimacy. However, the nature of advice may also be treated as a contextual factor in the design of SACs, and the extent to which the advice should be prescriptive may depend on the issue at hand. For example, if the SAC is asked to provide direct policy recommendations, some members of the panel will usually require expertise beyond the basic sciences to ensure the advice is relevant, legitimate, and of high quality.

### 3.7 Summary of Examples

SACs clearly vary with respect to sector, level, permanence, target audience, independence, and nature of advice. The variations along these six dimensions are all central features of the SAC landscape. **Table** **3** summarizes the characteristics of the example SACs discussed above.

**Table 3:** Characteristics of selected SACs

Name	Sector	Level of operation	Permanence	Target audience	Degree of independence	Nature of advice
IPCC	Environment	Intern.	Standing	External	Embedded	Descriptive
EASAC	Energy, biosciences, environment	Intern.	Standing	Internal	In‐between	Prescriptive
CCA	Inter‐disciplinary	National	Mixed	External	In‐Between	Descriptive
Puget Sound WQA	Environment	Sub‐national	Mixed	Both	Embedded	Descriptive
GESAMP	Environment	Intern.	Standing	External	Arms‐length	Mostly descriptive
STAP	Environment	Intern.	Standing	Internal	In‐between	Prescriptive
Defense Science Board	National security	National	Standing	Internal	Embedded	In‐determinate
Health Canada	Health	National	Ad‐Hoc	Internal	In‐between	Prescriptive
WBGU	Environment	National	Standing	Internal	Arms‐length	Prescriptive
UNSCEAR	Energy	Intern.	Standing	External	In‐between	Descriptive

## 4 Conclusion

This article outlines the current landscape of SACs and proposes a typology of SACs with the goal of assisting decision‐makers and researchers in designing and studying these committees. Our analysis demonstrates the wide variation in recent SACs. It also underscores how widespread SACs have become; SACs operate in nearly all sectors and at all jurisdictional levels. In outlining our typology, we have suggested how this tool can assist decision‐makers and researchers in considering the effectiveness of these committees.

While we have searched a wide range of literature to construct our typology, we did not conduct a full systematic review, which might have uncovered other typologies of which we are not aware. Finally, we have focused on SACs from Europe and the US; however, many examples of SACs from other regions could be highlighted. Despite these limitations, our typology is one of the most expansive to date, drawing from a wide variety of SACs in different countries and sectors.

Research on SACs is scarce relative to research on scientific advice more generally. The gaps in current knowledge are particularly pronounced when it comes to the institutional design of these committees and the determinants of their effectiveness. While outside the scope of our current analysis, the field would benefit from a better understanding of how factors, such as size, member composition, and decision‐making rule affect the effectiveness of SAC. For example, while many institutions have established procedures for securing diversity among members, there is little evidence on the impact of such diversity on the quality, relevance, and legitimacy of SACs. Moreover, there is a particular need for further research on SACs outside Europe and the US and in low‐ and middle‐income countries.

It is widely agreed that policy‐making should be informed by evidence and SACs have come to fill an important role in the science‐policy nexus. This article has outlined the global landscape of SACs and proposed a typology that can assist decision‐makers in designing SACs while helping expand the field and enabling researchers to study the elements of SAC design that make these committees most effective. This research can help ensure that the design of SACs themselves is guided by evidence, in an effort to bridge the broader gap between scholarly research and public policy.

## 5 Experimental Section

*Scoping Review*: A scoping review of the literature was conducted on SACs to provide a basis for the overview typology.^60^ The review aimed to identify examples of SACs and typologies of SACs. Both peer‐reviewed and gray literature were searched, as well as selected web pages affiliated with the scientific advisory committees were used as examples in the landscape and typology. Academic OneFile, EBSCOhost, Google Scholar, HeinOnline, JSTOR, ProQuest, Scholars Portal, and ScienceDirect were searched in the winter of 2016. It included a wide variety of terms whose meaning overlaps with “scientific advisory committee” and was conducted without date limitations. These searches were complemented with citation pearl growing–the use of characteristics of highly relevant publications to help identify other relevant publications–with particular emphasis on forward citations.

*Development of Typology*: A typology was developed from the results of the scoping review. Typologies classified entities into groups by similarities along two or more dimensions^61^ and were generally useful for reducing complexity and creating categories that allow for identification of similarities and differences.^62^, ^63^

The characteristics were selected to delineate categories based on two primary criteria. First, characteristics were favoured that helped describe the current landscape of SACs. Second, characteristics were favored that helped study the design of SACs with particular focus on the institutional determinants of their effectiveness. Note that the typology was not intended to be used to directly to evaluate SACs; rather the goal is a comprehensive description. That is, it is hoped that readers use this typology to guide them in understanding the association between the design and the effectiveness of SACs, rather than attempt to compartmentalize a given advisory committee into a rigid category.
